# A qualitative assessment of barriers and facilitators to implementing recommended infant nutrition practices in Mumbai, India

**DOI:** 10.1186/s41043-020-00215-w

**Published:** 2020-07-27

**Authors:** Priyanka Athavale, Kristin Hoeft, Rupal M. Dalal, Ameya P. Bondre, Piyasree Mukherjee, Karen Sokal-Gutierrez

**Affiliations:** 1 University of California, Berkeley, USA; 2 Department of Preventive & Restorative Dental Sciences and Center to Address Children’s Oral Health, University of California, San Francisco, USA; 3 Foundation for Mother & Child Health, 93/C Kamgar Nagar, SG Barve Marg, Kurla East, Mumbai, Maharashtra 400024 India; 4 New Horizons Health and Research Foundation (NHF), Unit No 10, Techniplex II, Veer Savarkar Flyover Bridge, Off S V Road, Opposite Hotel Grand Sarovar, Goregaon West, Mumbai, 400062 India; 5 Berkeley, School of Public Health, University of California, Berkeley, USA

**Keywords:** Malnutrition, Maternal behavior, Feeding methods, India, Infant nutritional physiological phenomena practices, Barriers, Complementary feeding

## Abstract

**Background:**

Childhood malnutrition has been a longstanding crisis in Mumbai, India. Despite national IYCF (Infant Young Child Feeding) guidelines to promote best practices for infant/toddler feeding, nearly one-third of children under age five are stunted or underweight. To improve child nutrition, interventions should address the cultural, social, and environmental influences on infant feeding practices. This study is an in-depth qualitative assessment of family barriers and facilitators to implementing recommended nutrition practices in two Mumbai slum communities, within the context of an existing nutrition education-based intervention by a local non-governmental non-profit organization.

**Methods:**

The population was purposively sampled to represent a variety of household demographics. Data were collected through 33 in-depth semi-structured interviews with caregivers (mothers and paternal grandmothers) of children age 0–2 years. Transcripts were translated and transcribed, and analyzed using qualitative analysis procedures and software.

**Results:**

A complex set of barriers and facilitators influence mothers’/caregivers’ infant-toddler feeding practices. Most infants were fed complementary foods and non-nutritious processed snacks, counter to IYCF recommendations. Key barriers included: lack of nutrition knowledge and experience, receiving conflicting messages from different sources, limited social support, and poor self-efficacy for maternal decision-making. Key facilitators included: professional nutrition guidance, personal self-efficacy and empowerment, and family support. Interventions to improve child nutrition should address mothers’/caregivers’ key barriers and facilitators to recommended infant-toddler feeding practices.

**Conclusions:**

Nutrition interventions should prioritize standard messaging across healthcare providers, engage all family members, target prevention of early introduction of sugary and non-nutritious processed foods, and strengthen maternal self-efficacy for following IYCF recommended guidelines.

## Background

Over one-third of child deaths worldwide occur due to undernutrition, with one-half of the child malnutrition deaths worldwide occurring in India, making childhood malnutrition an ongoing public health priority [1]. Child malnutrition results from a complex set of factors, including poor prenatal care, inadequate milk/food intake during infancy, and limited access to health services for both the newly born and the mother [2–5]. Children from birth to age two are most vulnerable because of their higher requirements for energy and nutrient-dense foods to support physical and mental development [6–9]. Malnourished children tend to have poorer performance in school and are more likely to grow into malnourished adults, and are at greater risk of disease and early death [10–12].

In India, the National Family Health Survey in 2015-16 found that 29% of urban children under 5 years were underweight and 31% were stunted, with children living in informal settlements or slums had higher rates of malnutrition than those in other urban areas [13]. Although the burden of child malnutrition has been declining since 1990 in India, it continues to remain the predominant risk factor for death in children in majority of states in India. According to the Lancet review of Child and Maternal Malnutrition in India, of the 1.04 million under-5 deaths in India, 68.5% can be attributed to malnutrition, with the prevalence of stunting and wasting at 39% and 33% respectively [14]. Few studies have documented the nutritional status of children living in informal settlements in Mumbai, Maharashtra, one of the largest urban cities in India. The National Family Health Survey (2014-2015) collected overall data from Maharashtra and reported that the rate of underweight children under 5 years was about 36%, with 38.4% stunted and 26.1% wasted respectively [13]. In terms of infant and young child feeding practices, the survey reported only 6.9% of breastfeeding children between 6-23 months living in urban neighborhoods in Maharashtra were receiving an adequate food intake; of non-breastfeeding children between 6-23 months, only 13% were receiving an adequate diet. Evidently, the underlying cause of persistent malnutrition in Mumbai and many other urban developments throughout India is due to poor feeding practices, along with several interrelated factors including maternal education, family support, availability and marketing of unhealthy snacks and structural barriers embedded within low socioeconomic status [15].

To address the gaps and challenges in addressing malnutrition as a public health issue in India, The Government of India and the Food and Nutrition Board and Ministry of Human Resource Development developed Infant and Young Child Feeding (IYCF) guidelines in 2004 to improve the nutrition of children from birth to 2 years [16, 17]. These include three main practices: continued breastfeeding or feeding with appropriate calcium-rich foods if not breastfed; feeding of solid, semi-solid, or soft food for a minimum number of times per day according to age and breastfeeding status; and inclusion of a minimum of three food groups per day according to breastfeeding status. While health workers may provide mothers information on IYCF, there are many individual, household, cultural, economic and environmental barriers to translating the information into practice [18]. Additionally, India has launched other various policy initiatives in the past two decades including the Integrated Child Development Scheme (1975), Mid-day Meal program in schoolchildren (1995) and the National Food Security Act (2013), but the prevalence of stunting, wasting and malnutrition remains persistently high. Studies have indicated that approximately 20% of under-five deaths could be prevented if all IYCF indicators were achieved and that 22% of neonatal deaths could be avoided with proper breastfeeding practices [15].

It is critical now, more than ever, to identify and address the barriers and facilitators to maternal caregiving practices as delineated in IYCF, especially in populations at high risk for malnutrition [19, 20]. While many studies have documented the gap and decline in improvement on nutrition parameters in India, none have outlined why elevated rates of malnutrition have persisted. Moreover, none have detailed the barriers to implementing IYCF practices in the context of a globalized food economy in which mothers and children are exposed to nutrient-poor and unhealthy snacks which compete with recommendations from IYCF [21]. Additionally, most infant/toddler nutrition studies inquire only about nutritious components of the IYCF guidelines and don't inquire about whether the children are fed non-nutritious snack foods and drinks. Among the quantitative studies that have inquired about non-nutritious snacks and drinks (e.g., Huffman, Bentley), they've found a very high prevalence of inappropriate feeding of non-nutritious snack foods and drinks to infants/toddlers. Qualitative studies are needed to better understand the personal, family, cultural and institutional contributors to non-nutritious feeding of infants/toddlers, and what key institutional supports are needed to ensure healthier infant/toddler feeding that follows the IYCF guidelines.

The primary aim of this study is to use qualitative methods to assess the underlying barriers and facilitators for caregivers to implement recommended infant and toddler feeding practices in two urban communities in Mumbai, India to augment existing IYCF-based interventions and help design sustainable and feasible interventional change.

## Methods

### Study design, population, and study setting

This study is a cross-sectional analysis of barriers and facilitators to implementing recommended infant-toddler feeding practices using qualitative methods. The method used in-depth interviews to deeply understand the causes of child undernutrition within the urban slum population in two specific slum communities, despite community-based counseling and education through healthcare facilities and local non-profit organizations. The study population is a convenience sample of low-to-middle income mothers and paternal grandmothers of children from 6-24 months of age living in two urban slum communities in Mumbai, India: Dhobi Ghat and Phule Nagar. These communities are informal settlements largely housing migrant families from rural areas. Most households consist of two parents with 1-3 children, many including grandparents and other relatives, living in a space of approximately 50-75 square feet. It is typical for family units to be joint families, consisting of the nuclear family living with the paternal grandparents of the child, and sometimes with the child’s aunts, uncles and cousins. The gender dynamic centered around males working day jobs and women primarily staying at home to care for children and complete household chores. Dhobi Ghat has a population size of approximately 100,000 and many adults are day laborers; Phule Nagar has a population of approximately 25,000 and most people are employed at a nearby university as custodial staff. Both sites have a similar level of sanitation and access to clean water, access to traditional foods and vegetables, lentils, rice, and access to modern non-nutritious snack foods and sugary sodas.

These communities were accessed through collaboration with the Mumbai-based non-governmental non-profit organization Foundation for Mother and Child Health (FMCH). Established in 2006, FMCH employs community health workers, social workers, nutritionists, child development specialists, nurses and physicians to provide free in-home and clinic-based interventions for pregnant women and young children to promote preventive health care, adequate nutrition and child development. Building on the principles of the IYCF guidelines, FMCH provides a pregnancy club with prenatal care, education, and support; child preventive health clinics with primary health care, dietary and growth monitoring; breastfeeding and weaning support; child development classes; cooking demonstrations; and specific nutrition guidance and supplementation based on children’s growth trajectory. At the time of this study, FMCH had been serving families in Dhobi Ghat for 10 years, and in Phule Nagar for 6 months.

### Participant eligibility and recruitment

Mothers were eligible to participate in the study if they had at least one child between the ages of 6 and 24 months, had attended and received IYCF counseling at an FMCH clinic at least once in the past 3 months, and spoke Hindi or Marathi.

**Fig. 1:**
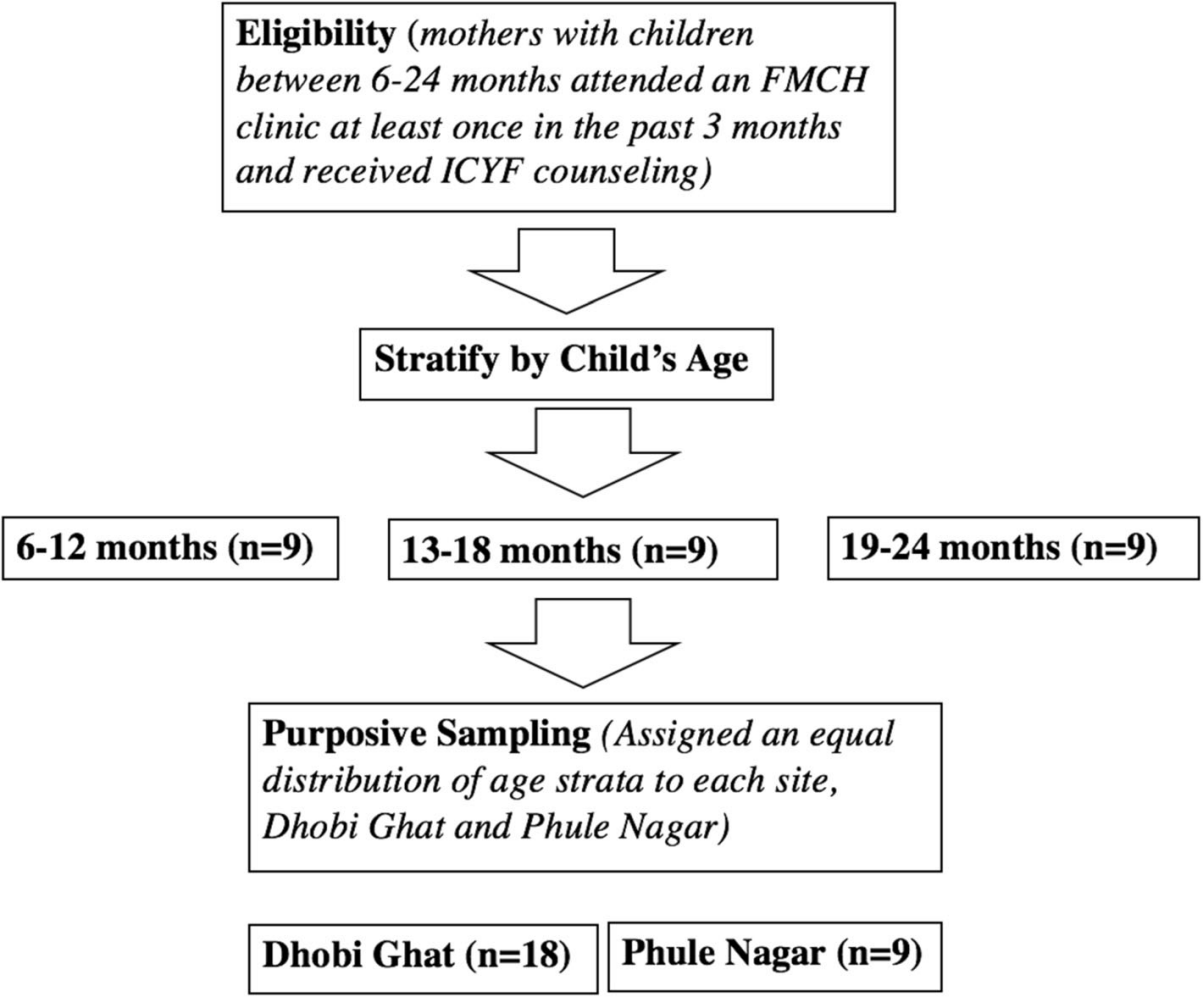
Sampling strategy for qualitative interviews with mothers

Participants were purposively sampled from both sites with equal sampling of families from three child age groups (6-12 months, 13-18 months, and 19-24 months) to ensure assessment of the specific feeding issues at each child age and developmental stage. All participants were recruited by a health worker from FMCH and first author (PA), and provided written and oral consent in their preferred language, either Hindi or Marathi. See Figure 1: Sampling strategy for qualitative interviews with mothers.

Participants were purposively sampled from both sites with equal sampling of families from three child age groups (6–12 months, 13–18 months, and 19–24 months) to ensure assessment of the specific feeding issues at each child age and developmental stage. All participants were recruited by a health worker from FMCH and first author (PA), and provided written and oral consent in their preferred language, either Hindi or Marathi (see Fig. 1: Sampling strategy for qualitative interviews with mothers).

### Data collection

**Fig. 2:**
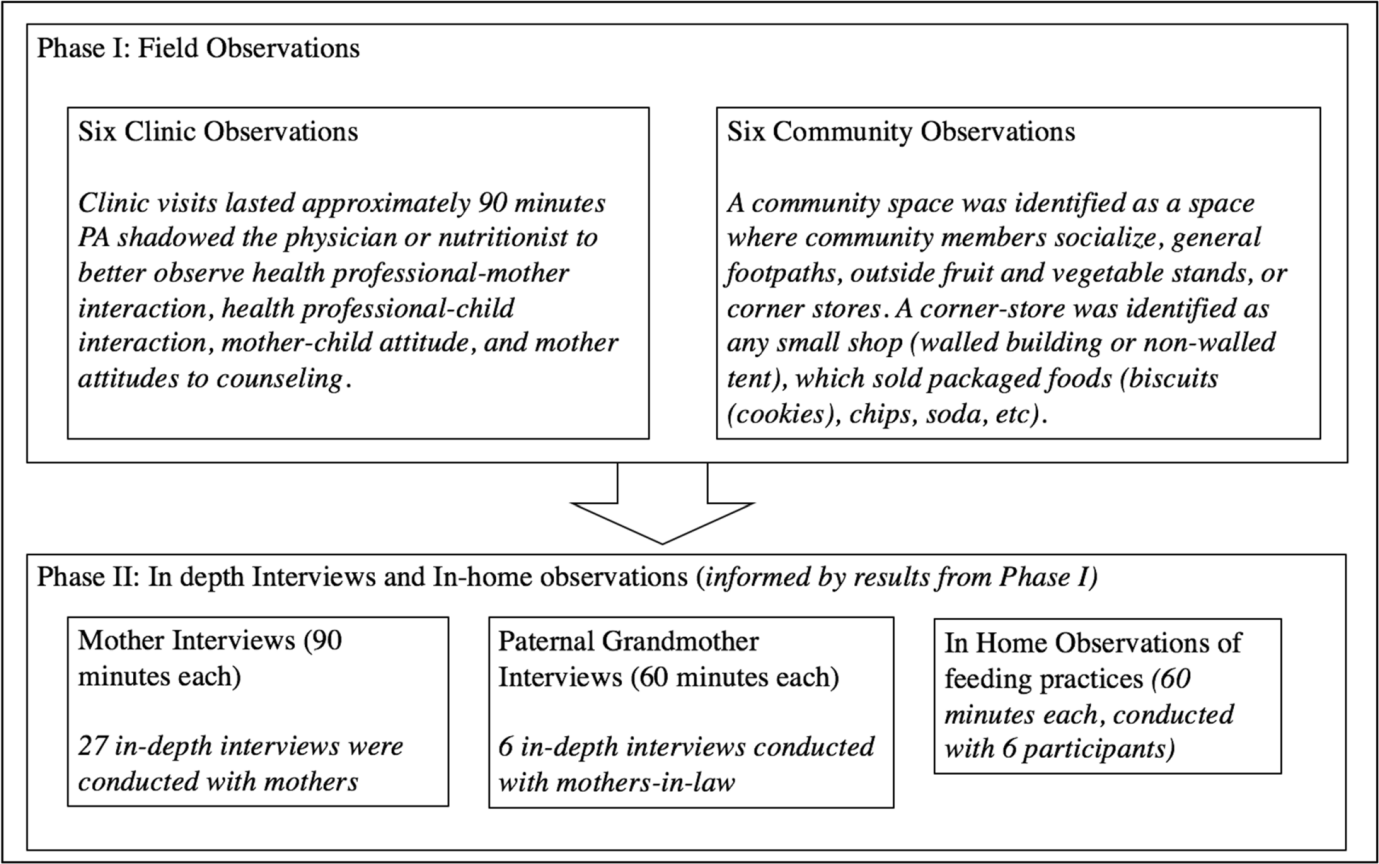
Flow chart describing study design flow: field observations, in depth interviews, and in home observations of feeding practices

Data were collected in two phases. During phase I, six 90-min field observations were conducted in community clinics and six in community spaces such as (vegetable and fruit stands, corner stores, taxi stands, and at intersections of main streets with side streets). Phase I results informed phase II instrument design and content. Note that this study does not report data on phase I, as it was merely used to inform phase II and interview design and questions. In phase II, 27 in-depth interviews were conducted with mothers and 6 in-home observations were completed of infant feeding practices during feeding periods. After approximately 10 interviews, it was clear that mothers-in-law had a significant impact on the mother’s caregiving abilities and child’s nutrition. As a result, 6 in-depth interviews with paternal grandmothers were conducted to better understand family dynamics and influence on maternal practices. Data were collected between November 2014 and January 2015. The interviews were conducted by PA in the participant’s home, in the participant’s preferred language, Hindi or Marathi. Each in-home observation was 60 min long, and each interview lasted 60–90 min. Figure 2 (flow chart describing study design flow: field observations, in depth interviews, and in home observations of feeding practices) depicts the research process through phase I and phase II.

### Interview and observation instruments

Based on review of the literature and extensive participant observations and field notes in FMCH community clinics and public spaces in both communities, a semi-structured interview guide and an observation guide were developed and implemented in phase II. The topics for interviews of mothers and paternal grandmother and for in-home feeding observations are seen in Table 1.

**Table 1:** Interview and observation components

Mother and paternal grandmother interview	• Childbirth: mental and physical experience • Breastfeeding: time of initiation, exclusivity, attributes to breast milk, breastfeeding management • Complementary feeding: initiation of complementary feeding, beliefs about complementary foods, snack food consumption, variety of foods given to child, complementary feeding management • Responsive feeding: feeding situation, caregiver response to child behavior during feeding, hunger cues, child independence in feeding • Organizational influence: compliance with FMCH recommendations, general feedback to FMCH intervention and services
In-home feeding observations	• Hygienic practices observed: hand washing with soap, washing dishes with soap, cleaning utensils and dishes before serving, sanitary disposal methods • Meal preparation: who prepared meal, time to prepare meal; mother’s emotional tone while preparing meal • Feeding situation: who is feeding child, placement of child during meal, child independence while eating, texture and consistency of food, caregiver’s interaction with child during feeding

### Data management and analysis

In-depth interviews with caregivers were translated and transcribed in English, and transcripts were thematically analyzed using NVivo and Excel, using inductive coding to identify emerging themes. First, key interview quotes and observations were sorted by topic, then a second round of coding was completed to identify key barriers and facilitators for proper infant and young child feeding practices within each topic and across multiple topics. PA coded the data with regular consultation with coauthors to refine codes. Analysis was complete once coding had reached saturation. Institutional Review Board (IRB) approval was received from University of California, Berkeley (approval #2012-11-4798) and written approval was provided by the Board of Directors at FMCH.

## Results

### Family demographic characteristics

Thirty-three in-depth interviews (27 mother interviews and 6 paternal grandmothers) were completed. Mothers had an average age of 26 years, and 9 years of schooling. Half of the mothers lived in a joint family, including extended family members such as paternal grandparents, aunts, and uncles. Over half of the families (54%) had a monthly income of less than 10,000 INR per month (less than $140 USD/month or $5-7/day). Children’s mean birthweight was 2.75 kg. When the mother first received FMCH services, the children’s average age was 8 months, and at the time of this study, children’s mean age was nearly 16 months, yielding an average of 8 months of FMCH service to date. Both sites had similar family demographic characteristics (see Table 2).

**Table 2:** Child and family demographic and nutrition characteristics (*n* = 27)

Maternal and household characteristics	% or mean ± SD (range)
Mother age (years)	25.6 ± 3.56 (21–24)
Mother education (years)	8.7 ± 4.55 (0–15)
Monthly income	
< Rs. 5000–Rs. 10,000	54%
Rs. 10,000–Rs. 15,000	32%
> Rs. 15,000	16%
Living in joint family (%)	50%
Number of children	1.7 ± 1.29 (1–5)
Child characteristics	
Average child age (months)	15.6 ± 2.25 (6–24)
Average birthweight (kg)	2.8 ± 1.25 (2.5–3.9)
Age at first visit to FMCH (months)	8 ± 2.52 (2–14)
Average number of months receiving care from FMCH	7.6 ± 3.8 (2–20)
Infant-toddler feeding practices	
Breastfeeding within 1 h of delivery	52%
Exclusive breastfeeding for 6 months	33%
Child nutrition status (at time of interview)	
Stunting	50%
Underweight	33%
Wasting	17%

Qualitative analysis of the in-depth interviews resulted in 6 core themes, which were categorized into barriers and facilitators. The main themes and patterns are described below, with representative quotes that illustrate each theme.

### Barriers to implementing recommended IYCF practices

#### Lack of knowledge and experience

Most mothers received prenatal care and delivered their babies in large public hospitals. Many, especially first-time mothers, reported feeling unprepared for their delivery, post-partum care, and breastfeeding, and felt that the hospital staff was too busy to advise or help them. Additionally, 10 of the 27 mothers (37%) had unplanned caesarean sections, and many felt physically impaired afterwards. One mother stated:

After the delivery the doctor didn't tell me anything. They just discharged me immediately the next day.

While all mothers had attended at least one FMCH session, most had limited prior experience with breastfeeding. For the purposes of this paper, “exclusive breastfeeding” will be defined according to WHO and UNICEF guidelines as early initiation of breastfeeding within 1 h of birth and exclusive breastfeeding for the first 6 months of life with introduction of nutritionally adequate and safe complementary (solid) foods at 6 months together with continued breastfeeding up to 2 years of age or beyond [22]. Only 14 of the 27 mothers (52%) initiated breastfeeding within the first hour of delivery, as recommended by Infant and young child feeding. (n.d.-b) and FMCH. After delivery, many mothers were unaware of the normalcy of low-volume colostrum in the first few days, blamed themselves for their inability to produce breastmilk, and felt too ashamed to ask hospital staff for advice. A mother explains:

The nurse said that after 1 hour, I needed to feed [the baby] breast milk. ... After my delivery, I wasn't getting milk … I felt that it was my first time, and I didn't even know, and I didn't have the courage to ask the doctors. So I just sat there. I did try to breastfeed her, but my milk wasn't reaching her mouth.

The mothers’ uncertainties included how often and how long to breastfeed; when to start solid foods, what foods, quantity, and frequency; and how to recognize their baby’s hunger cues, and encourage their child’s independence during feeding. Many mothers simply made the best decisions they could without professional guidance. Some mothers over-fed and some under-fed their infants. Some continued exclusive breastfeeding past 6 months, and others fed their babies the same solid foods (typically mashed rice and lentils) for every meal, rather than a variety of foods differing in texture, color, flavor, and nutrient type, which is what the rest of the family would typically eat for meals. One mother explained that she started feeding her 2-month-old infant cow’s milk:

No one really told me [what to do]. The neighbors would tell me that cow’s milk is good; goat’s milk is good—it’s good for small children, that’s what they say. For the formula, you make it, and then when it’s used up we can’t immediately afford it either. So that is why we started cow’s milk [after he turned 2 months].

In contrast, some mothers actively solicited information or received advice from family members, friends, community resources, and health professionals. When the advice from different sources was consistent, mothers tended to follow it; however, often the advice was inconsistent and confusing, as discussed below.

#### Receiving conflicting information from different sources

Many mothers received information from multiple sources including family members (particularly mothers-in-law, fathers-in-law, brothers- and sisters-in-law), neighbors, corner-store owners, mothers of similar-aged children, health workers, nurses, pharmacists, and physicians. Often, recommendations from these many sources were contradictory.

One of the more difficult contradictions for mothers to navigate was between healthcare professionals and their family. Although most mothers understood their healthcare providers’ recommendations for exclusive breastfeeding, they had difficulty translating their knowledge into practice when their family provided conflicting recommendations. Most mothers were inclined to follow the advice of their family and community elders—since this was their cultural tradition, and they needed to live with and depend upon them on a daily basis. Many mothers were strongly influenced by their mothers-in-law, particularly in the post-partum/newborn period when they were feeling tired. One mother said:

Whom should I listen to- my mother-in-law or FMCH? That’s how I feel.

While all 27 mothers were counseled by FMCH about exclusive breastfeeding for the first 6 months, 18 out of 27 mothers (67%) reported feeding their infant other liquids or food during this period. In both sites, it was widely believed that breast milk did not contain sufficient nutrients for a growing infant for 6 months, and that babies needed additional nutrients. Sometimes, parents felt additional nutrients came from packaged biscuits and formula, as they were advertised for having key nutrients and vitamins. In one interview, the brother in-law contributed to the conversation about packaged foods. Both the paternal grandmother and brother-in-law explained that they started giving the infant chocolate and other solid foods before 6 months:

Yeah, we [brother-in-law and paternal grandmother] gave it to him to try. If he doesn't eat chocolate and biscuits, then where will he get the strength? Milk doesn't have any nutrients.

For babies born prematurely, low birthweight, or with other medical problems, family members were even more likely to advise against exclusive breastfeeding and to start solid foods earlier, as a mother recounts:

When she [my baby] was 2 months, my mother-in-law would crush some Parle G [sweet biscuits containing flour, sugar, salt and preservatives] in milk and feed it to her. Because when she was born, she was only 1 kg … she was very delicate, so we were stressed.

The inconsistency in information led mothers to feel uncertain about whose advice to follow, and anxious about their own caregiving abilities.

#### Limited social support

Many mothers felt that they lacked adequate social support—i.e., trusted people to advise, assist and comfort them—particularly during their labor and delivery experience, starting breastfeeding, and initiating solid foods for their infant. Some mothers had few or no family members to rely on, as they had immigrated to the city from their family village.

Although half of the mothers lived in joint family households, this did not guarantee social and emotional support. Mothers living in large extended families—particularly with their husband’s family and paternal grandmother—described complex family dynamics, additional household responsibilities, limitations in their ability to make decisions, and stress from family quarrels, as one mother describes:

We were all living together in one house. My brother-in-law’s 4 children, and all of their wives and all that... it’s a lot of pressure. On top of that, my mother-in-law and father-in-law are old so they weren’t able to properly take care [of themselves] at that time... I was very stressed.

In addition, most of the extended family members, including fathers and grandmothers were also working full time, leaving the mother alone to care for her newborn and older children.

While the mothers received informational support from healthcare providers at FMCH and clinics, most expressed the need for more emotional and social support to implement the recommended practices. Several mothers reported having a dominant secondary caregiver (such as a sister-in-law or mother-in-law) who undermined their attempts to cook different dishes at home or limit their child’s snack food consumption. Often, the mother’s overwhelming feeling of stress and self-doubt compounded with the power imbalance led her to yield the decision-making authority to her mother-in-law.

#### Low perceived maternal self-efficacy for decision-making

Many mothers expressed concerns about their inability to put into practice their knowledge and desires for their baby’s health and nutrition (low self-efficacy). This theme emerged throughout all four stages: delivery, breastfeeding, complementary feeding, and responsive feeding. Many mothers lacked confidence in their own decision-making ability and sought approval—often from their mother-in-law—prior to making a decision regarding their child’s nutrition and health. Mothers with poor self-efficacy feared that their child would become ill, and they would be blamed, if family members’ and elders’ advice was not followed. One mother explains:

I don’t really listen to [FMCH]. If anything, I have to listen to my mother-in-law you know. I told them [mother-in-law] that FMCH tells me something different, but then she tells me that she raised 5 kids- and [I] have only one- what do I know? She thinks that I don’t know what to feed my child and what not to feed.

Early in infancy, during the period of recommended exclusive breastfeeding, many mothers felt anxious that their breast milk was inadequate, and fed their babies additional formula, cow’s milk, and solid foods such as sweet biscuits softened in milk or water, contrary to recommendations. When transitioning to complementary feeding, most mothers were reluctant to introduce a variety of foods and try new recipes for their baby, fearing that their baby would not like or accept the new food, that the new food would make their baby sick, and that their family members would criticize them. For example, some mothers were hesitant to introduce vegetables since they had harder texture and could choke the child. Many mothers-in-law perpetuated these fears. One mother explained the challenges of trying variety in complementary foods for her 5-month old child:

When he gets older–like when he’s the age of eating these things [vegetables]– then, I will start them. Now, he is young and I don’t know if he will eat what I make. So why make something new right now?

In approximately two-thirds of the families interviewed, the mother said that her mother-in-law was the primary decision-maker regarding family meals. Most mothers reported that they were unable to decide what food to prepare for their child because their mother-in-law, husband, or father-in-law went to the market to purchase the ingredients. A mother explained:

My mother-in-law does everything. I only make the food. My mother-in-law handles what to buy. She decides everything.

Mothers also reported having little control over their children’s snacks. Although FMCH physicians and dieticians strongly advised mothers against giving their children non-nutritious snacks (e.g., chocolate, candy, biscuits, and chips), most mothers reported that their children frequently consumed these unhealthy snack foods, and that they were usually purchased and introduced by other family members (mother-in-law, father-in-law, sister-in-law, brother-in-law, father), elderly neighbors, or neighborhood children. While all the mothers understood that processed and non-nutritious snack foods were unhealthy for their babies, mothers felt uncomfortable educating elders in their family and community what they had learned. The mothers’ status in the family, and their fear of disrespecting elders, outweighed their ability to implement the experts’ recommendations for caring for their child’s nutrition and health.

### Facilitators in implementing recommended IYCF practices

#### Professional nutrition guidance

Mothers who received support from FMCH and attended clinics with the nutritionist and pediatrician consistently benefitted from their counseling. One mother who was an attendee of FMCH clinics reports:

I learned to breast feed every 2 hours from the doctor in the FMCH clinic, they told me to feed her every 2 hours, and how to feed her milk. They showed me all that.

Another mother states:

Since I was pregnant, I’ve been going to FMCH and I’ve been attending all their

sessions… My aunt told me that I might not be making enough breastmilk. Then I asked the doctor in FMCH, and she said no- don’t think like that. She told me not to stress and this is normal and natural

One to one counseling sessions at FMCH with a professional who is able to reassure the mother of her practices, are critical to improve the mother’s self-efficacy and confidence in trying new methods, engaging with nutrition recommendations for her infant, and addressing family and community perceptions around breastfeeding and complementary feeding.

#### Personal self-efficacy and empowerment

Personal self-efficacy and self-confidence emerged as strong themes in enabling mothers to follow experts’ recommendations and overcome household and community challenges in caring for their infant. Mothers with more innate confidence and self-efficacy prioritized following health professionals’ advice, which they believed was salient for their child’s health. Majority of these mothers were consistent with breastfeeding exclusively for 6 months, appropriately introducing a variety of healthy complementary foods and preventing their children from eating unhealthy snack foods. Self-efficacy was a critical facilitator in empowering the mother to overcome her lack of experience, conflicting advice and opinions, uncertainty and fear about not meeting her mother-in-law’s expectations, and pervasive consumption of snack food in the community. They were more likely to stand up to pressure from unsupportive family members, neighbors, community members, and the advertising messages. A mother describes her experience:

Everyone tells me to start [child, 6 months old] on biscuits and crackers. I get into arguments all the time with my in-laws about it. They told me to give him biscuits for a meal. And I told them, who knows when the biscuits were made, where they come from. We have no idea about what’s even in them. We shouldn’t give children biscuits from such a young age. But then my father-in-law tells me, other children [in the community] have biscuits and their health is fine, so then why should it be bad for [child]?! They keep telling me to dip a biscuit in milk and give to him. But I am firm- I said no, I will not give him.

#### Family support

Mothers who spent time in their maternal home—as opposed to their mother-in-law’s home—during the third trimester of pregnancy, prior to delivery, reported feeling emotional and instrumental support. Similarly, mothers who returned to their maternal home after delivery, for a resting period of 1–2 months, reported substantial family support. During this period, mothers were advised to rest completely while the immediate or extended family members cared for their baby and household chores.

While breastfeeding early on, mothers who received family support in massaging the breast and helping the baby to latch on felt more comfortable and self-assured in their technique. Despite breastfeeding being time-consuming, mothers who had help at home felt that it was possible to breastfeed while managing other household responsibilities.

Mothers who had family support from their own mother or an unusually positive relationship with their mother-in-law tended to have positive attitudes about their childcare abilities, desire to follow the recommendations received from the nutritionist and pediatrician, interest in trying new things, determination to carry out best practices, and persistence in the face of challenges. They were more likely to exclusively breastfeed for 6 months, introduce solid foods at the appropriate time, try a variety of foods, and continually reintroduce recommended foods even if the baby initially rejected them. These mothers also preferred feeding their babies home-cooked dishes, and were more likely to avoid giving their children junk food. A supportive mother-in-law was especially important when the mother had other barriers such as lacking experience, hesitant to try new things, and had low self-confidence in her caregiving abilities.

## Discussion

This study reveals a web of complex factors that influence a mother’s capacity to implement recommended nutrition practices, as delineated by the IYCF guidelines.

The major barriers to proper infant-toddler feeding were experienced at the start (i.e., with early and exclusive breastfeeding) and with the expansion of feeding (i.e., the transition to complementary feeding). At both stages, many mothers experienced barriers to implementing the guidelines: low knowledge, received conflicting information, poor self-confidence, and experienced limited support from family members [23–25].

Breastfeeding was inhibited by physical constraints after a caesarean section, lack of information and support to start breastfeeding right after delivery, and inability to ask hospital staff for help. Many mothers thought that they could not produce enough breastmilk, and introduced cow’s milk, biscuits, and chocolate in early infancy, and results were consistent with breastfeeding barriers identified by Parekh and colleagues which was conducted in Mumbai, India [26]. Researchers at KEM Hospital in Mumbai in 2004 interviewed 99 women to evaluate newborn feeding practices post-delivery, concluding that women should receive information regarding proper IYCF practices during the antenatal period [27–29]. While antenatal counseling is essential, our findings also suggest that mothers need unified messaging regarding appropriate practices and strong social support–before and after delivery–to facilitate optimal breastfeeding [30].

Exclusive breastfeeding for 6 months and proper complementary feeding were negatively influenced by the widespread advertising of processed snack foods (like Parle-G biscuits, salty snacks such as Kurkure, candies, chocolates, sweet desserts, and sugary drinks), and encouragement by family and community members to make such snacks a regular part of young children’s diet which supports reports that the nutrition transition is rampant in India [31–33]. In 2002, Engle et al studied barriers to proper infant and young child feeding in India, and found that women delayed introduction of complementary foods [34]. However, since 2002, the influx of western foods, packaged foods, and sweets in urban India has led to our findings that complementary foods such as biscuits dipped in milk are started before 6 months, and as early as 2-3 months of age [35]. Huffman et. al recently used Demographic and Health Survey Data to determine the consumption of processed snack foods in 18 low-and middle-income countries. Although this analysis did not include India, the study reported that up to 68% of children 6-23 months old in Asian datasets consumed sugary snacks [21]. Bentley et all further substantiates this data and reports that sugary snacks were consumed by up to 74% of children in the same age range and savory snacks by 57% [15]. This data is consistent with the qualitative data we present, suggesting that sugary and savory snacks pose a great threat to appropriate complementary feeding in infants, with perhaps a greater risk in the poorest communities where snack foods are easy to access and marketed as being “healthy”. Moreover, these early feeding practices could set the precedent for later food preferences which can contribute to the double burden of stunting and overweight children in India. To address these challenges, several studies have reported successes of interventions targeting complementary feeding practices [36–40]. Bhandari et. al. studied a complementary feeding program in rural Haryana for infants ages 6-18 months, concluding that an educational intervention could improve complementary feeding practices if it addressed factors that influenced feeding, such as gender bias, hygiene in the home, and exclusive breastfeeding prior to complementary feeding [41]. Despite the development of several new interventions in the past decade, barriers to complementary feeding in India still exist today and many children suffer from inappropriate complementary foods due to lack of maternal knowledge, conflicting information from various sources like family and media, and individual-level barriers such as self-efficacy.

Major themes from the mothers’ narratives can be used to focus and redesign interventions targeted towards mothers and family members in India to improve infant and child feeding practices. Table 3 describes key barriers and implications for interventions within an urban slum population in India to improve IYCF outcomes.

**Table 3:** Implications and recommendations from findings for programmatic interventions

*Barrier*	*Recommendation for interventions*
Lack of knowledge and experience in recommended IYCF practices	Improved counseling for mothers and family members regarding IYCF practices to increase knowledge and confidence of mothers’ to implement recommended guidance
	National policies to limit marketing of non-nutritious processed foods to enable good nutrition during pregnancy and early childhood
Receiving conflicting information from different sources	Include the mother’s mother-in-law in discussion regarding infant nutrition and health is critical-through home visits, grandmother groups, and counseling in dyads for mothers and their mother-in-laws.
	Standardized training on IYCF practices for healthcare professionals to minimize conflicting messages and confusion.
Limited social support and low self-efficacy for decision-making around childcare and nutrition	Emphasis on women’s empowerment and community-development to improve women’s self-efficacy

## Limitations and strengths

There are several strengths and limitations of this study. In terms of limitations, generalizing the findings to mothers outside of the organization (FMCH) in which the study was conducted has limited validity in the context of this study. Additionally, some interviews were very hard to conduct confidentially with the mother—many families lived in small quarters and mother-in-laws, other household members (aunt, uncle, sister, neighbors) would step in and out of the home during the interview. This could compromise the openness of the interviewee and responses, despite the precautions of confidentiality taken by the researcher. Another primary limitation was that the interview quality and style was heavily dependent on one interviewer (PA). A single researcher provides consistency across interviews; however, question order and content could be influenced by the researcher’s biases and preferences. Finally, the researcher’s presence, which is unavoidable during qualitative data gathering can often influence the subject’s responses.

Despite the above limitations, there are several strengths of this study. The nature of qualitative methods and design allowed for richness and detail through the in-depth interviews with mothers and mother-in-laws. These interviews were pivotal in elucidating key barriers and limitations to proper maternal and child nutrition in our study population and without the person-to-person interaction, several dynamics and behaviors which contribute to barriers to implementing IYCF would not be revealed. The iterative nature of the methods allowed for addition of new interviews with mother-in-laws which added richness and a new perspective to the study aims. Moreover, the study design, specifically being housed within a non-profit organization, allowed for clear and prompt feedback to the community. The study participants are all beneficiaries of FMCH and receive maternal and child prenatal and postnatal care from this non-profit organization. This allowed for the study to take place in a uniquely situated population—one that was receiving IYCF counseling—where study results could directly inform interventional change through FMCH.

## Conclusion

Narratives from the interviewed participants suggest that a complex interaction between barriers and facilitators affect adherence to IYCF guidelines. Barriers and facilitators crossed multiple developmental feeding stages––exclusive breastfeeding and introduction of complementary foods. Our data suggests that community health organizations should re-emphasize the IYCF guidelines, target and prevent the early introduction of sugary, non-nutritious processed foods, ensure standard messaging across all healthcare providers, engage extended family members in addition to mothers, and make efforts to bolster mother’s self-efficacy in child nutrition counseling. Such strategies can overcome the barriers to proper IYCF and can build upon on the facilitating factors to improve maternal-child and family health.

## Data Availability

The datasets used and/or analyzed during the current study are available from the corresponding author on reasonable request.
